# A Roadmap for Using Hybridisation Capture–Based Target Enrichment of Ancient Environmental DNA in Palaeoecology

**DOI:** 10.1111/1755-0998.70152

**Published:** 2026-06-08

**Authors:** Nicole R. Foster, Luke E. Holman, Linda Armbrecht, Jérémy Courtin, Theis Jensen, Mikkel Winther Pedersen, Lennart Schreiber, Hannes Schroeder, Frederik V. Seersholm, Giulia Zampirolo, Kristine Bohmann, Heike H. Zimmermann

**Affiliations:** ^1^ Centro de Estudios Avanzados de Blanes Consejo Superior de Investigaciones Cientificas Blanes Spain; ^2^ Section for Molecular Ecology and Evolution Globe Institute, University of Copenhagen Copenhagen Denmark; ^3^ Institute for Marine and Antarctic Studies, University of Tasmania Hobart Tasmania Australia; ^4^ Australian Centre for Excellence in Antarctic Science (ACEAS) Institute for Marine and Antarctic Studies, University of Tasmania Hobart Tasmania Australia; ^5^ Geological Survey of Denmark and Greenland Department of Glaciology and Climate Copenhagen Denmark; ^6^ Section for GeoGenetics, Globe Institute University of Copenhagen Copenhagen Denmark

**Keywords:** aDNA, ancient eDNA, bait capture, eDNA, hybridisation capture, metagenomics, probes, sedaDNA

## Abstract

Recovering ancient DNA from environmental samples is transforming the way we understand historical ecosystems. While high‐throughput sequencing of the total DNA in environmental samples (shotgun metagenomic sequencing) reveals the taxonomic contents of these samples, the genetic signals of some taxa (e.g., eukaryotes) can be weak compared to the background levels of DNA from organisms such as bacteria, requiring deep sequencing approaches that are costly. Thus, to increase cost‐effectiveness, pre‐sequencing enrichment of target DNA can be advantageous. One technique to enrich this target DNA is hybridisation capture, where short RNA or DNA baits are designed to match, bind and isolate specific stretches of DNA. Hybridisation capture has previously been applied to recover DNA from ancient skeletal remains, but it is only beginning to emerge as an approach to characterise organisms from ancient environmental samples. Thus, there is limited information on establishing hybridisation capture workflows for ancient environmental DNA applications, including the limitations and advantages. This mini review focuses on establishing a roadmap for the applications of hybridisation capture to ancient environmental DNA samples.

## 1 Introduction

Environmental DNA (eDNA) is the DNA shed by organisms into the environment (soil, sediment, air and water), and this can be harnessed to assess the biological composition of environmental samples (Deiner et al. 2017). When this eDNA becomes incorporated into stratified archives such as ice, sediment and permafrost, it can be preserved for decades to millions of years, forming a biological time capsule. Analysis of this now ancient eDNA enables palaeoecological reconstructions and insights into long‐term ecosystem responses to environmental change (Armbrecht et al. 2022; Foster et al. 2020; Kjær et al. 2022; Pedersen et al. 2015; Thomsen and Willerslev 2015; Willerslev et al. 2003).

Ancient eDNA can be analysed using shotgun metagenomic sequencing or DNA metabarcoding. For shotgun metagenomics, a comprehensive taxonomic profile can be retrieved by randomly sequencing the pool of extracted eDNA without focusing on a specific genetic marker (Quince et al. 2017). This approach preserves the characteristic fragment length distributions and damage patterns of ancient DNA, such as short fragment lengths and cytosine deamination, which are critical to discriminate between ancient and modern DNA (Armbrecht, Hallegraeff, et al. 2021; Orlando et al. 2021). It also reflects the uneven distribution of DNA sequences from different taxa in an environmental sample. This means it can underestimate or fail to recover genetic information from taxa present in low abundance, such as eukaryotes, that can represent less than 1% of the total sequences in an environmental sample (Stat et al. 2017). As an example, Armbrecht et al. (2024) showed that a shotgun metagenomic sequencing approach failed to recover sequences for the harmful algal bloom *Noctiluca scintillans* in marine sediment samples, while a targeted approach to detect this species in the same sample was successful. Increasingly deeper sequencing can improve the recovery of taxa making up only a small proportion of the sequencing pool; however, this greatly multiplies costs and computational requirements to process the sequences. Enriching the DNA of target taxa prior to sequencing can be a solution to maximise the proportion of DNA sequences of interest.

DNA metabarcoding is a widely used enrichment approach that amplifies target genetic regions using primers and polymerase chain reactions (PCR). This process relies on the PCR amplification and sequencing of variable DNA regions, which are flanked by conserved regions used for primer binding. As only the DNA marker of interest is sequenced, metabarcoding represents a cost‐effective approach to recover DNA for community assessments (Taberlet et al. 2012). Multiple metabarcoding markers have been used to characterise the taxonomic composition of environmental samples (Stat et al. 2017), ranging in size from ~10 to 143 bp (e.g., *trnL*; Taberlet et al. 2007) or 76 bp (e.g., *diat‐rbcL*; Stoof‐Leichsenring et al. 2012) and longer fragments up to ~400 bp (e.g., 18S‐V4; Comeau et al. 2011). Marker choice depends on the taxonomic group of interest, which can be broad, for example, eukaryotes (Amaral‐Zettler et al. 2009; De Schepper et al. 2019) or more selective, for example, fish (Miya et al. 2015; Holman et al. 2025), plants (Kress and Erickson 2007; Pedersen et al. 2013), foraminifera (Pawłowska et al. 2014, 2020; Nguyen et al. 2023) or diatoms (Armbrecht, Eisenhofer, et al. 2021; Buchwald et al. 2024; Zimmermann et al. 2021). Particularly, group‐specific markers, such as the diatom‐specific *diat‐rbcL*, typically result in a much better taxonomic resolution than what can be obtained through shotgun metagenomics (Armbrecht, Eisenhofer, et al. 2021; Buchwald et al. 2024; Zimmermann et al. 2021). Furthermore, the dependence of metabarcoding on specific gene regions allows for simple bioinformatics workflows.

Nevertheless, metabarcoding can induce biases due to preferential amplification arising from primer mismatches, meaning not all taxa are amplified equally. This is exacerbated with ancient eDNA, where degradation often produces fragments < 100 bp, which is shorter than most standard metabarcoding genetic regions (Kjær et al. 2022; Pedersen et al. 2015). This decreases the likelihood that both primer binding sites will be intact for successful PCR amplification, leading to a biased representation of the sample community (Armbrecht, Eisenhofer, et al. 2021; Deagle et al. 2014; van der Loos and Nijland 2021). Smaller gene regions are therefore preferred for ancient eDNA studies; yet, this can reduce the taxonomic resolution (Knapp and Hofreiter 2010; Zimmermann et al. 2024). Furthermore, metabarcoding obscures the original DNA fragment length variability and damage patterns because it selectively amplifies a fixed genetic marker, producing uniformly sized amplicons that limit authentication of ancient eDNA.

**FIGURE 1:**
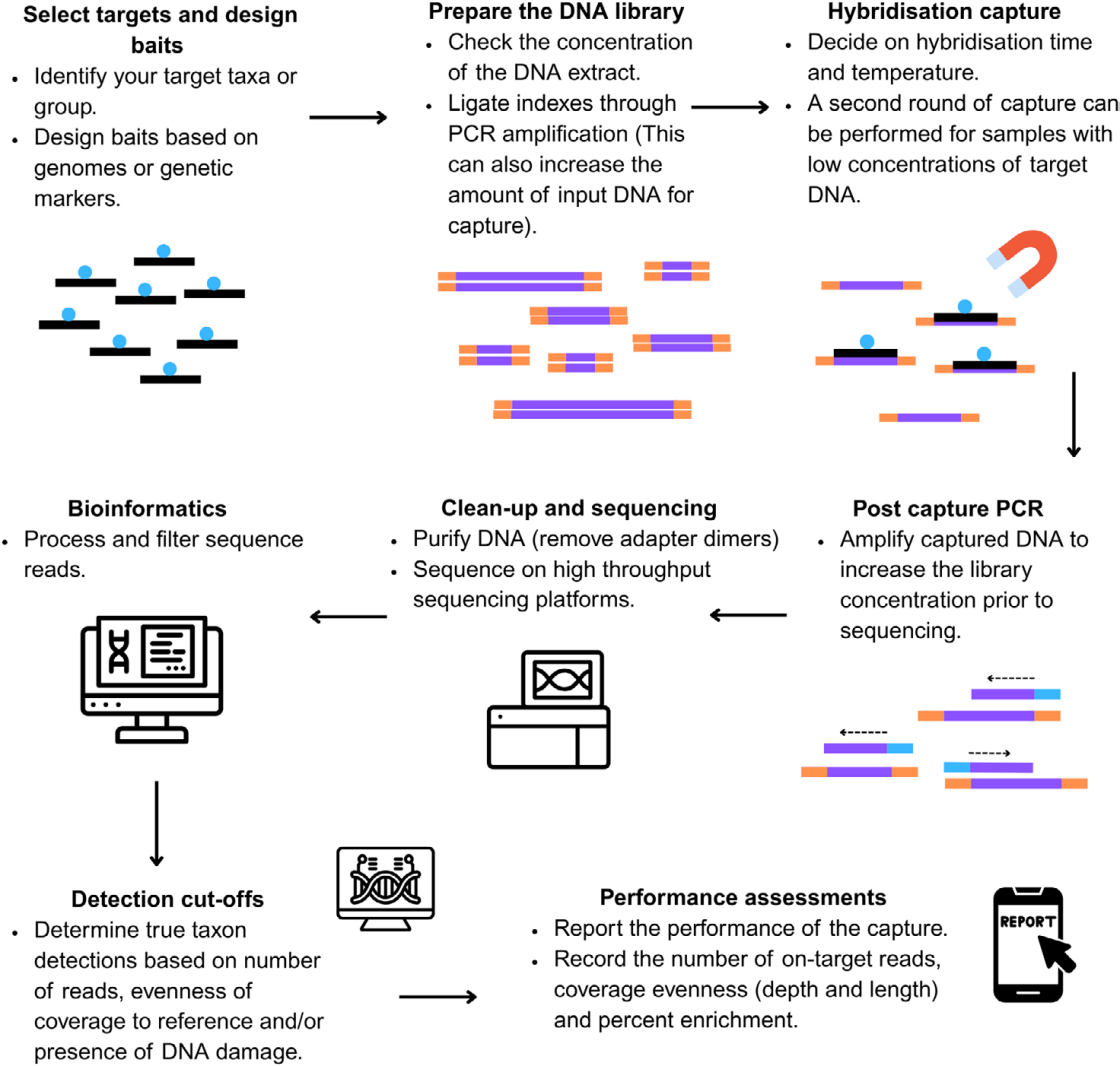
An overview of the laboratory and analytical steps to undertake hybridisation capture projects on ancient eDNA.

Hybridisation capture (also known as targeted capture) provides an alternative enrichment approach (Figure 1) to recover target DNA sequences while preserving DNA damage patterns (fragmentation and chemical damage) for ancient eDNA verification (Armbrecht, Hallegraeff, et al. 2021). This method builds upon shotgun metagenomic sequencing, but prior to sequencing the total DNA pool of a sample, target DNA fragments are ‘captured’ using specially designed baits (or ‘probes’) that hybridise (bind) with complementary target DNA, and the bait/DNA complex is then isolated and sequenced to increase the proportion of on‐target DNA sequences. This approach showed effective recovery of target sequences from ancient skeletal remains characterised by low endogenous DNA content—typically < 1% of the total DNA content (Carpenter et al. 2013; Templeton et al. 2013). It is therefore emerging as an effective approach for enriching DNA from ancient environmental samples when metabarcoding or shotgun metagenomic sequencing is ineffective (Ledger et al. 2025; Murchie, Kuch, et al. 2021). While hybridisation capture has been successfully applied to modern eDNA samples and in clinical contexts (see Bravo et al. (2025), for a comprehensive review), this mini review focuses on the emerging applications of hybridisation capture to ancient environmental samples within palaeoecology. We present a roadmap for undertaking hybridisation capture projects on ancient eDNA, including bait design, wet lab procedures and data analysis, outlining limitations, advantages, best practices and considerations.

## 2 Recommendations for Undertaking Hybridisation Capture on Ancient Environmental Samples

### 2.1 Target Choice and Bait Design

#### 2.1.1 Target Choice

Selecting appropriate target taxa is a critical first step in hybridisation capture studies of ancient environmental samples. This decision should consider the expected taxa within the study region, the quality of the DNA (i.e., how degraded it is), the genetic region(s) to target, the expected target DNA abundance (rare vs. dominant taxa) and the availability of reference sequences. When all of this is considered, baits can be designed and synthesised or generated directly from species of interest (Carpenter et al. 2013; Maricic et al. 2010; Schmid et al. 2017; Schulte et al. 2021; Templeton et al. 2013). Longer targets are suggested to stabilise the bait‐target hybridisation reaction (Suchan et al. 2022), acknowledging that, in ancient samples, targets are usually 100 bp or less.

#### 2.1.2 Bait Design

In hybridisation capture projects, baits can either be single‐stranded (RNA) or double‐stranded (DNA) and can be applied on both an array (Okou et al. 2007) and in solution (Gnirke et al. 2009). Array capture relies on the immobilisation of baits to a solid surface that then allows hybridisation of the target DNA, and unwanted DNA sequences are washed away. For in‐solution capture, baits are biotinylated and added to the target DNA in solution to allow for hybridisation between the bait and target DNA. The bait/DNA complex is then isolated using magnets, leaving behind non‐target DNA. In‐solution capture is the most commonly used approach for ancient eDNA, likely due to the improved recovery of target DNA for smaller gene regions compared to array capture (Mamanova et al. 2010).

Designing baits to capture target DNA is dictated by the research question, that is, whether the aim is to capture gene regions to discern phylogenetic relationships or to recover presence‐absence information for species or communities. Baits can be designed to capture specific target gene regions or whole genomes. It is recommended that baits are at least 50 bp long to ensure stable hybridisation between the target DNA and the baits (Nota et al. 2024; Suchan et al. 2022). Many ancient eDNA studies employ baits that are ~80 bp in length (e.g., Armbrecht, Hallegraeff, et al. 2021; Foster et al. 2024), but longer baits have also been successfully applied to recover target DNA fragments that are shorter than the baits (Schulte et al. 2021, 2022). Multiple overlapping baits should be generated for each target gene region, for example, starting a new bait every 20 nucleotides, with a threefold overlap recommended to increase coverage and maximise the recovery of ancient eDNA (Cruz‐Dávalos et al. 2017).

Research questions of a phylogenetic nature require baits spanning multiple taxa, which can necessitate large bait sets that can be costly; on the other hand, smaller bait sets can introduce capture bias as efficiency decreases with increasing genetic divergence between the bait and target (Glenn and Faircloth 2016; Jones and Good 2016). This can be overcome by designing baits complementary to ultra‐conserved elements, which allows capture across divergent taxa while recovering flanking variable regions that can differentiate between taxa (Lemmon et al. 2012; McCormack et al. 2012). Baits designed to capture these regions have generated data to resolve deep phylogenies based on the slow‐evolving rate of these regions and resolve shallow phylogenies based on the recovery of flanking variable regions (Jones and Good 2016). However, these regions that flank ultra‐conserved elements are not always sufficiently variable, limiting their use to differentiate closely related taxa (Glenn and Faircloth 2016).

To recover informative variable regions to discern closely related taxa or populations, baits can instead target conserved exons, including those that encode proteins (Glenn and Faircloth 2016; Li et al. 2013) and standard metabarcoding loci (e.g., 18S, trnL; Günther et al. 2022; Krueger et al. 2021; Murchie, Kuch, et al. 2021). This approach involves compiling reference sequences for selected gene regions, aligning these and then generating a bait set to capture across the length of these sequences. Baits designed to capture metabarcoding gene regions can benefit from existing reference databases and the typically well‐established bioinformatic workflows (Holman et al. 2023). However, targeted capture of degraded eDNA may not always recover the entire target gene region, potentially hindering accurate assignment of DNA sequences to modern references. In this case, it is best to design baits across multiple metabarcoding regions (which can also help resolve taxonomy for groups without a universal gene region, such as plants; Foster et al. 2022) or choose shorter regions that are more likely to be intact in ancient environmental samples.

Designing baits to capture multiple gene regions increases the number of baits that need to be synthesised and, by extension, the cost; however, degenerate bait sets can be produced based on bait sequence similarity thresholds or clustering to reduce redundancy and the number of baits required (Krueger et al. 2021; Lentz et al. 2021; Murchie, Kuch, et al. 2021). For example, Armbrecht, Hallegraeff, et al. (2021) designed a bait set based on assembled reference sequences and then removed baits with > 83% overlap and > 95% identity, decreasing the bait kit size. In bait design, it is also important to ensure that the baits do not bind to non‐target sequences to prevent unwanted hybridisation. Given hybridisation can occur with sequences that have a > 70% match, baits with this similarity to non‐target sequences should be removed (Alanko et al. 2022). Additionally, baits can be queried against non‐target reference sequences and/or genomes as a proxy for whether off‐target hybridisation will occur, and any baits that return hits should be removed.

Reduced bait sets can also be designed using ancestral sequence reconstruction, where phylogenetic trees are constructed, and baits are designed on selected internal nodes. For land plants, nodes were selected where the tips have at most 9% nucleotide dissimilarity to the most distal ancestral sequence, maximising taxonomic breadth while minimising bait redundancy (Nota et al. 2024). This leverages the fact that baits can tolerate some sequence divergence—up to 25%–30% under permissive capture conditions (i.e., one mismatch between the bait and target DNA every four to five bases; Delsuc et al. 2016; Mariac et al. 2018; Nota et al. 2024). This means that baits can be used to capture regions of shared ancestry (homologous regions), leading to capture across multiple descendant species (Delsuc et al. 2016; Dickson et al. 2021; McLay et al. 2021; Waycott et al. 2021). This can be particularly useful when capturing species that have gone extinct, as baits can be designed on modern relatives (Delsuc et al. 2016; Pipes and Nielsen 2022; Slon et al. 2022).

### 2.2 Bait Synthesis and Software

Numerous software programs are available to assist with bait design, including: BaitsTools (Campana 2018), Baitfisher (Mayer et al. 2016), CATCH (Metsky et al. 2019), eProbe (Huang et al. 2024), HUBDesign (Dickson et al. 2021), MetCap (Kushwaha et al. 2015), MrBait (Chafin et al. 2018), ProbeTools (Kuchinski et al. 2022), Syotti (Alanko et al. 2022) and SupeRbaits (Jiménez‐Mena et al. 2022). Alternatively, the design can be outsourced and/or premade bait sets purchased through companies that also synthesise them, including Agilent (USA), IDT (USA), Twist Bioscience (USA), and Daicel Arbor Biosciences (USA). Baits can also be generated from PCR‐amplified DNA derived from specimen samples, where the amplicons are then biotinylated (Adams et al. 2024; Maricic et al. 2010; Schulte et al. 2021). These PCR products can also be reverse transcribed into RNA baits for use in capture (Richards et al. 2019; Snyder‐Mackler et al. 2019), or RNA baits can be produced by implementing RADseq on messenger RNA to obtain a reduced representation of the target gene region (Schmid et al. 2017). While RNA baits exhibit stronger binding power and greater capture of AT regions within sequences, DNA baits are better at capturing high GC regions (Zhou et al. 2021). Therefore, it is recommended that RNA baits be designed to capture target DNA that has a GC content between 30% and 60% (Chilamakuri et al. 2014; Nota et al. 2024). In these instances, RNA baits have a greater binding affinity and stability than DNA baits, which leads to a higher capture efficiency and more uniform recovery of degraded DNA from ancient environmental samples (Furtwängler et al. 2020).

### 2.3 Wet Lab Procedures

#### 2.3.1 Library Preparation

Preparing the DNA library for typical capture projects involves estimating the concentration and the fragmentation of the DNA extract. Sonication or enzymatic fragmentation is undertaken to reduce the size of the DNA fragments to enable hybridisation with the baits; however, in ancient eDNA studies where the DNA is already shortened through degradation, sonication can lead to over‐shortening of the target DNA fragments and reduce capture success (Foster et al. 2024). Therefore, we advise not to sonicate if the DNA is expected to be highly degraded. The concentration of the input DNA library can also impact capture success, where recommendations range from a minimum of 100 ng (Arbour Biosciences 2019) and 10–200 ng (Agilent Technologies 2016). However, as little as 10 ng of target DNA can be enough for capture in clinical applications (Agilent Technologies 2016), and in ancient environmental samples (marine sediments), target capture has been successful with as little as 50 ng of input library DNA (Armbrecht, Hallegraeff, et al. 2021).

#### 2.3.2 Indexing

A first amplification occurs as part of the library preparation (prior to capture) and uses primers designed to match sequencing adapters, combined with single or dual indexes. The indexed libraries allow samples to be pooled prior to capture, which in some cases can increase the amount of input DNA for hybridisation. Post‐capture PCR employs primers designed to amplify the sequencing adapter and index combination and is undertaken to ensure sufficient concentration of the captured target DNA for sequencing. However, this can increase the likelihood of inter‐sample chimaeras in the sequencing step, that is, when two or more DNA templates are incorrectly joined together, resulting from incomplete primer extension during PCR. This can lead to sequences being assigned to the wrong samples. To avoid this, dual indexing should be used, ideally unique ones, to enable estimation of index jumping rates, that is, misassigned sequences due to incorrect index incorporation (Kircher et al. 2012). Additionally, amplification cycles should be optimised to prevent over‐amplification.

#### 2.3.3 The Capture Reaction

Capture success depends on creating optimal conditions for the baits and target DNA to hybridise. This includes considering the GC content and length of the bait and the hybridisation time and temperature. Longer baits and higher GC content increase the stability of the hybridisation reaction and generally improve specificity (reduced off‐target binding) relative to shorter baits, as more base pairs are required to hybridise for stable binding (Gasc et al. 2016). However, longer baits can tolerate a greater number of mismatches, which can facilitate the capture of more divergent targets. This can be enhanced by using lower hybridisation temperatures, for example, at 55°C (Schreiber et al. 2025). Conversely, implementing higher hybridisation temperatures can improve stringency, for example, 65°C (Daicel Arbor Biosciences 2020), and reduce off‐target binding. Increasing the specificity of bait capture by using higher temperatures can also be combined with longer durations (24–48 h) (Cruz‐Dávalos et al. 2017; Paijmans et al. 2016). However, this can lead to evaporation of the sample, an increase in non‐specific annealing and DNA degradation (Daicel Arbor Biosciences 2020). To mitigate the latter effects, hybridisation can be performed for a few hours at a high temperature, then at a reduced temperature for the remaining time (Murchie, Monteath, et al. 2021; Armbrecht et al. 2024). In ancient eDNA studies where the DNA is short, shorter baits (~80 bp) can be more effective at capturing this DNA, where higher hybridisation temperatures are recommended to decrease non‐specific binding and maintain capture efficiency.

In addition, specificity can be increased through an additional round of capture on the first captured library (Li et al. 2013), either using the same bait set or a different one. Of course, this will double the reagents, costs and time of the capture step. Other means of improving capture efficiency are the addition of blockers, which are short regions of DNA designed to bind to unwanted genetic regions to prevent capturing non‐target DNA, such as repetitive regions (Ávila‐Arcos et al. 2011) or adapter sequences. The latter is a common technique, with suitable blockers usually being provided with commercial bait kits. As mentioned briefly above, performing hybridisation capture on a multiplexed library as input is a frequently used technique to save costs and time. However, using single‐sample libraries as input for capture increases the likelihood of hybridising to the target DNA and is most useful in ancient eDNA studies where the targets are a small proportion of the total DNA (Zavala et al. 2022). Overall, a systematic study examining the optimal hybridisation conditions for ancient environmental samples is urgently needed.

#### 2.3.4 Post‐Capture Processing

Sample clean‐up and sequencing are the final steps in the hybridisation capture workflow. It is important to remove adapter‐dimers (formed when two adapters ligate to each other instead of to the DNA template) before sequencing, which can be performed using magnetic beads, gel‐excision or Pippin Prep/BluePippin (Sage Science). The latter offers the most accurate size selection method, yet is the most expensive and can result in loss of the library (between 5% and 95% from personal observations). Gel excision is a cheaper option, but this can be labour‐intensive. Magnetic bead‐based DNA purification is a fast option, but may not provide as accurate removal of adapter/primer dimers as the other approaches. Optimising the amounts of sequencing adapters, primers and polymerase can help to reduce unwanted adapter or primer‐dimer formation.

Illumina sequencing is commonly used for studies performing capture on ancient eDNA, where reads are typically very short. The required sequencing depth and coverage across target gene regions depend on the research question and budget, with greater coverage necessary to assess phylogenetic relationships and reduce sequencing error. However, increasing sequencing depth across the target gene regions also increases the costs of sequencing, so this needs to be weighed against the goals of the capture project.

### 2.4 Data Analysis and Reporting

#### 2.4.1 Data Analysis

Bioinformatic approaches to analyse the sequence data generated from hybridisation capture projects depend on the study design and research question. Often, hybridisation capture data is processed similarly to shotgun metagenomic sequencing data, including quality control and filtering, mapping of reads to reference sequences and then filtering taxonomic assignments using lowest‐common‐ancestor (LCA) approaches based on defined sequence similarity thresholds (e.g., Murchie, Monteath, et al. 2021; Vernot et al. 2021). Several bioinformatics programs have been established (e.g., PHYLUCE: Faircloth 2016; HybPiper: Johnson et al. 2016) and can be used and/or integrated depending on the specific research goals (e.g., species identification [HybPiper] vs. phylogenomics [PHYLUCE]).

Detection cut‐off frameworks can increase certainty around detections from ancient environmental samples. These have been established for eDNA metabarcoding data but are often based on the occurrence of taxonomically assigned DNA sequences across PCR replicates (Alberdi et al. 2018), and this approach cannot be applied to hybridisation capture data. Instead, taxonomic assignments are commonly filtered by the presence of DNA damage to authenticate ancient DNA (Gelabert et al. 2025; Zavala et al. 2021) or evenness of coverage across the target gene regions (as opposed to reads clustering on conserved regions; Vogel et al. 2023) and minimum read thresholds, below which taxonomic detections are discarded (Gelabert et al. 2021). Negative controls containing no target DNA template can be used to guide the determination of minimum read thresholds, where the number of reads should be close to zero, and this is important to assess potential contamination.

#### 2.4.2 Reporting

Performance assessments are important when reporting on ancient eDNA capture projects, for example, when applying FAIR standards to eDNA assays (Takahashi et al. 2025). As these have not yet been established for hybridisation capture projects, we recommend reporting (i) the number of reads aligning to the reference sequences used to design the baits as a measure of capture specificity (this can be achieved using recommended strict mismatching parameters; for example, BWA‐aln allows for mismatches of 1% [−n 0.01] of the sequence length with two maximum insertions or deletions [−o 2] Oliva et al. 2021), (ii) the percentage of on‐target reads (reads recovered for the target taxa), including the depth and length of coverage of target genetic regions/genomes and (iii) the percent enrichment, for example, the percentage of the target gene region or genome that was enriched (García‐García et al. 2016; Papaiakovou et al. 2025). Reporting the number of reads mapping to target taxa can be used as an indicator of off‐target recovery and can be standardised as a percentage of the total reads; it can also be used to establish read thresholds for assigning taxon presence by comparing to negative controls. The coverage evenness, that is, depth and length of the target DNA regions that are recovered, can be another indicator of capture efficiency and identify any biases in capture, that is, regions of low or high GC content. The per cent enrichment, defined as the proportion of on‐target reads, can be used to assess the effectiveness of the hybridisation capture for comparing to shotgun metagenomic sequencing and metabarcoding.

## 3 Applications of Hybridisation Capture to Ancient Environmental Samples

In the past decade, hybridisation capture has advanced the reconstruction of ancient environments by enabling the recovery of near‐complete genomes of plants and animals from ancient sediments. One way this has been done is to implement biotinylated long‐range PCR products from modern species to capture ancient sequences. For example, Schulte et al. (2021) amplified the chloroplast genome of *Larix* ( *L. gmelinii* and *L. sibirica* ) using long‐range PCR, then biotinylated the PCR products and used these to recover the near‐complete chloroplast genome of *Larix* from 6700‐year‐old sediments. Here, degraded sequences were recovered from across the genome and then mapped bioinformatically to reassemble the chloroplast genome. Compared to shotgun metagenomic sequencing, Schulte et al. (2021) achieved a 155‐fold increase in on‐target reads and could resolve this species' range shifts over the past 30,000 years (Schulte et al. 2022). Similarly, Gelabert et al. (2021) used biotinylated long‐range PCR amplicons from the modern mitochondrial genomes of humans and canids to successfully enrich these ancestral genomes from ~25,000‐year‐old cave sediments.

Genome‐scale information can also be retrieved from ancient sediments by collating reference genomes and synthesising baits to capture these. Several studies have implemented this approach to isolate human mitochondrial and nuclear DNA from ancient sediments (Zavala et al. 2021; Zhang et al. 2020), which facilitated the recovery of haplogroup information that was not detected with shotgun metagenomic sequencing (Sawafuji et al. 2025) and has been shown to recover informative single‐nucleotide polymorphisms preserved in 200,000‐year‐old sediments (Vernot et al. 2021). This approach has even recovered whole human mitochondrial genomes from sediments that matched the haplotype of nearby skeletal remains, despite DNA concentrations in the sediment being two orders of magnitude lower than the human remains (Sarhan et al. 2021). This highlights the capacity of hybridisation capture to extract genetic information from organisms that are not preserved physically (as fossils) in environmental samples (Massilani et al. 2022). Similarly, baits designed to capture mammal mitochondrial genomes found DNA from leopards, foxes and mammoths in 30,000‐year‐old sediments where there were no physical remains (Gelabert et al. 2025). Further, baits designed from archived mammoth genomes were used to capture a near‐complete ancient mammoth genome from faecal remains despite the high level of background DNA from bacteria and the mammoth's dietary taxa (Karpinski et al. 2017).

Capture approaches have been further implemented to understand ancient ecosystems. For example, Murchie, Monteath, et al. (2021) designed baits to capture Arctic flora and fauna using ~180 megafauna mitochondrial genomes and ~2100 plant taxa across metabarcoding loci *matK, rbcL* and *trnL*. Applying this to 30,000‐year‐old sediments, they discovered an ecosystem shift around 13,500–10,000 years ago, revealing a change from a steppe‐tundra ecosystem to a woody shrub ecosystem. Similarly, Kjær et al. (2022) employed the same bait set to detect ancient communities in 2‐million‐year‐old sediments in Greenland, discovering the presence of ancient reindeer and mastodon DNA, and concluded that their site had a higher historical productivity and habitat biodiversity than previously thought. Similarly, in an archaeological context, Lentz et al. (2021) designed baits to target nine chloroplast gene regions using references for flora known to be historically present in the study area. They applied this bait set to capture plant DNA from sediment samples recovered from ancient Mayan reservoirs and were able to identify native tropical flora instead of the hypothesised domesticated plants.

There have been several instances where capture‐based approaches were the only way genetic information of target taxa could be acquired from ancient environmental samples. In the example above, Murchie, Monteath, et al. (2021) showed that their bait set targeting Arctic flora and fauna was able to capture up to 10.8% on‐target sequences, whereas shotgun metagenomic sequencing recovered only 0.007% on‐target sequences and no ecologically informative data. Similarly, Ledger et al. (2025) collated mitochondrial and nuclear gene regions from extant parasite DNA reference sequences and designed baits to capture these regions. They applied this to sediment samples collected from human settlement sites in the Neolithic and Roman periods and could identify several species and genera of ancient parasites, whereas shotgun metagenomic sequencing detected no parasite DNA. This highlights that hybridisation capture can yield target DNA that is inaccessible using shotgun metagenomic sequencing without the use of expensive deep sequencing (Ledger et al. 2025). However, the studies described above used baits that were designed to target specific taxa, and it remains unclear whether universal bait sets can be designed to capture across broad groups of organisms, for example, all eukaryotes.

## 4 Limitations and Future Work

Hybridisation capture presents a promising approach to recover target DNA from ancient environmental samples that are present in low concentration and so cannot be amplified with metabarcoding or are not recovered from shotgun metagenomic sequencing. However, one of the main limitations to undertaking hybridisation capture projects is the resources required (e.g., laboratory equipment and reagents) and associated costs. Costs for capture projects can exceed $120 per sample, but this varies greatly with the bait design, that is, the number of baits required to achieve successful capture of the target DNA regions. Current capture protocols also use many reagents, require specialised equipment and expertise and are time‐consuming to implement. Nevertheless, protocols for automating the process and multiplexing samples are emerging to reduce both costs and time (Zavala et al. 2022).

Beyond improvements in deep sequencing technologies, other emerging technologies are likely to enhance enrichment methods in the future. Novel technologies such as CRISPR, a gene editing technology, may allow us to ‘cut‐away’ target DNA (Quan et al. 2019), creating an alternate enrichment technique that would require less specialised equipment and therefore reduce costs. These applications are beginning to emerge in the field of eDNA (Kardailsky et al. 2025; Littleford‐Colquhoun and Kartzinel 2024; Phelps 2019), but there are many unknowns so far, and no studies on ancient eDNA have employed these methods. As we work towards improving the techniques to enrich and recover target DNA from ancient environmental samples in the most accurate and reliable way, we will begin to unlock the full potential of ancient eDNA.

## Funding

Open Access funding provided thanks to the CRUE‐CSIC agreement with Wiley. This study was supported by the European Union's Horizon Europe Marie Sklodowska ‐Curie Actions, 101105307 (POSIDONIArXiv); the European Research Council (ERC) under the European Union's Horizon 2020 Research and Innovation Programme (grant agreement no. 856488); the Independent Research Fund Denmark (DFF FunCap; https://doi.org/10.46540/2098‐00026B); and the Australian Research Council (ARC) Discovery Projects DP250100886 and DP250103420.

## Conflicts of Interest

The authors declare no conflicts of interest.

## Data Availability

Data sharing is not applicable to this article as no datasets were generated or analysed during the current study.
